# MiR-7 reduces the BCSC subset by inhibiting XIST to modulate the miR-92b/Slug/ESA axis and inhibit tumor growth

**DOI:** 10.1186/s13058-020-01264-z

**Published:** 2020-03-06

**Authors:** Miao Li, Meng Pan, Chengzhong You, Fengshu Zhao, Di Wu, Mei Guo, Hui Xu, Fangfang Shi, Danfeng Zheng, Jun Dou

**Affiliations:** 1 Department of Pathogenic Biology and Immunology, School of Medicine, Southeast University, 87 Ding Jiaqiao Rd., Nanjing, 210009 China; 2 Jiangsu Province Hospital, the First Affiliated Hospital of Nanjing Medical University, Nanjing, 210029 China; 3 Department of General Surgery, Zhongda Hospital, School of Medicine, Southeast University, Nanjing, 210009 China; 4 Department of Gynecology & Obstetrics, Zhongda Hospital, School of Medicine, Southeast University, Nanjing, 210009 China; 5 Department of Oncology, Zhongda Hospital, School of Medicine, Southeast University, Nanjing, 210009 China

**Keywords:** Breast cancer, miR-7, Cancer stem cells, Subset, lncRNA XIST

## Abstract

**Background:**

Breast cancer stem cells (BCSCs) are typically seed cells of breast tumor that initiate and maintain tumor growth. MiR-7, as a cancer inhibitor, decreases the BCSC subset and inhibits tumor progression through mechanisms that remain unknown.

**Methods:**

We examined miR-7 expression in breast cancer and developed a BCSC-driven xenograft mouse model, to evaluate the effects of miR-7 overexpression on the decrease of the BCSC subset in vitro and in vivo. In addition, we determined how miR-7 decreased the BCSC subset by using the ALDEFLUOR, lentivirus infection, dual-luciferase reporter, and chromatin immunoprecipitation-PCR assays.

**Results:**

MiR-7 was expressed at low levels in breast cancer tissues compared with normal tissues, and overexpression of miR-7 directly inhibited lncRNA XIST, which mediates the transcriptional silencing of genes on the X chromosome, and reduced epithelium-specific antigen (ESA) expression by increasing miR-92b and inhibiting slug. Moreover, miR-7 suppressed CD44 and ESA by directly inhibiting the NF-κB subunit RELA and slug in breast cancer cell lines and in BCSC-driven xenografts, which confirmed the antitumor activity in mice injected with miR-7 agomir or stably infected with lenti-miR-7.

**Conclusions:**

The findings from this study uncover the molecular mechanisms by which miR-7 inhibits XIST, modulates the miR-92b/Slug/ESA axis, and decreases the RELA and CD44 expression, resulting in a reduced BCSC subset and breast cancer growth inhibition. These findings suggest a potentially targeted treatment approach to breast cancer.

## Introduction

Breast cancer is often treated successfully with surgery and conventional therapies; however, approximately 25% of patients eventually develop treatment resistance and recurrence of metastatic disease, which is a major cause of mortality in women worldwide [1]. Studies have demonstrated that the cancer stem cells (CSCs) are typically seed cells of tumors that initiate and maintain tumor growth, and traditional chemotherapy is usually able to shrink the tumor mass, but often fails to completely eradicate CSCs [2–4].

Accumulating evidence suggests that noncoding microRNA (miR-7) is a breast cancer suppressor because it inhibits Kruppel-like factor-4 (KLF-4), which is one of the oncogenes that enhance breast cancer growth [5–7]. High expression of miR-7 significantly reduced both the capacity of self-renewal and invasion of breast cancer stem-like cells (BCSCs). Interestingly, the long noncoding RNA (lncRNA), terminal differentiation-induced lncRNA (TINCR), is required for normal epidermal differentiation by a posttranscriptional mechanism [8]. Recently, a report showed that TINCR modulated KLF4 expression by competing with miR-7, to mediate its oncogenic function [9, 10]. MiR-7 can downregulate SET domain bifurcated 1 (SETDB1), an oncogene that is overexpressed in breast cancer, resulting in the inhibition of tumor progression and metastasis in mice, accompanied by a decrease in the BCSC subset in tumor tissues, as in our previous report [6]. The exact molecular mechanism, however, has yet to be elucidated.

Therefore, the present study aimed to further investigate the mechanisms of miR-7-mediated reduction of the BCSC subset and elucidate its molecular linkage in breast cancer cells and in breast cancer xenografts in mice.

We report that lncRNA XIST (X-inactive-specific transcript), which is involved in genomic imprinting and the transcriptional silencing of genes on the X chromosome and is highly expressed in tumor tissues [11–15], is an epigenetic regulator targeted by miR-7 that directly bound and inhibited XIST expression in breast cancer by using the RNA in vivo precipitation (RIP) method, which underlies the attenuation of properties of BCSCs and the decrease of the BCSC subset. MiR-7 also modulated the miR-92b/Slug/ESA (epithelium-specific antigen) axis and reduced CD44 and Slug expression, resulting in a decrease in cancer cell stemness and the BCSC subset in breast cancer. Furthermore, miR-7 overexpression could suppress the tumorigenicity of CD44^+^CD24^−^ESA^+^BCSCs and reduce the BCSC subset in breast cancer animal model. Our study characterizes the regulation of XIST by miR-7 and provides evidence that overexpression of miR-7 or downregulation of XIST underlies the attenuation of properties of BCSCs and the decrease of the BCSC subset, which may be a potential strategy for breast cancer therapy.

## Materials and methods

### Cell culture

Human breast cancer SK-BR-3, MCF-7, and MDA-MB-231 cells were obtained from the Cellular Institute in Shanghai, China. The LD cell line was established by our lab from a human breast cancer postsurgery sample that was cultured and characterized as shown in Additional file 1: Figure S1. SK-BR-3 and MCF-7 cells were maintained in 1640 medium. MDA-MB-231 and LD cells were cultured in DMEM.

### Human breast cancer samples

Human breast cancer postsurgery samples were obtained from the Department of General Surgery of Zhongda Hospital at Southeast University in China. The investigation was approved by an ethics committee at Southeast University School of Medicine, and informed consent for the use of the postsurgery samples was obtained from the donors who were breast cancer patients. The clinical data of 12 breast cancer patients and specimens are shown in Additional file 4: Supplementary Table S1.

### RT-qPCR

Reverse transcription quantitative real-time PCR (RT-qPCR) analyses were used and performed on an ABI StepOnePlus Real-Time PCR System. Total cellular RNA was isolated from each sample by using a Qiagen RNeasy Kit (Qiagen, Valencia, CA). One microgram of total RNA per sample was subjected to cDNA synthesis using the Superscript III Reverse Transcriptase (Invitrogen). The mRNA levels of the genes of interest were expressed as the ratio of each gene of interest to β-actin mRNA per sample. cDNAs were amplified by PCR with primers as listed in Additional file 4: Supplementary Table S2 [16].

### Western blotting

Cells and tumor tissue homogenates were lysed in protein extraction buffer (Novagen, Madison, WI, USA) according to the manufacturer’s protocol. Briefly, proteins (10 μg/lane) were electrotransferred onto a polyvinylidene difluoride membrane, after which the membrane was blocked with 4% dry milk in Tris-buffered saline with Tween-20 for 1 h and then incubated with a rabbit antibody specific to human Slug (Affinity, Cincinnati, OH, USA), ESA (Proteintech, Chicago, USA), NF-κB subunit RELA (Santa Cruz Biotechnology, CA, USA), or CD44 (Proteintech, Chicago, USA) overnight at 4 °C. The membrane was rinsed for 5 min with an antibody wash solution 3 times before adding goat anti-rabbit fluorescently labeled secondary antibody (LI-COR, Lincoln, USA) overnight at 4 °C. Immunoreactive bands were detected by Odyssey scanning instrument (LI-COR Odyssey Imaging System, USA) [17].

### MACS for the preparation of BCSCs

CD44/CD24 and CD44/CD24/ESA antibodies conjugated to magnetic microbeads (Miltenyi Biotec, Bergisch Gladbach, Germany) were used to obtain BCSCs from the MDA-MB-231 cell line. The sorting method followed the manufacturer’s instructions and our previous study. We named CD44^+^CD24^−^ or ESA^+^CD44^+^CD24^−^ cells BCSCs [18–20].

### Plasmid construction and dual-luciferase reporter assays

We used the miRcode algorithm (release 6.2, http://www.mircode.org/) to search for miR-RNA targets of XIST, RELA, and Slug. Wild-type/mutated XIST, RELA, and Slug were generated by PCR from human genomic DNA. For the luciferase reporter assay, the PmirGLO Dual-Luciferase miRNA Target Expression Vector was used [6, 16]. The oligonucleotide sequences (wild-type) were used are shown in Additional file 4: Supplementary Table S3. Luciferase reporter assays were performed using the Dual-Luciferase Reporter Assay System (Promega) [21, 22].

### Oligonucleotide and plasmid transfection

Oligonucleotides were purchased from GenePharma (Shanghai, China). MiR-7 mimic, miR-92b mimics, and negative controls (Applied Biosystems) were used in gain-of-function experiments. The XIST siRNA, RELA siRNA, and negative control siRNAs were used in loss-of-function experiments (Additional file 4: Supplementary Table S4) [10, 16, 23].

### Infection with lentivirus encoding miR-7 vector

Lentivirus encoding miR-7 or empty vector was used to infect MDA-MB-231 or LD cells as previously described [6]. The clones with stable miR-7 expression were selected by GFP expression.

### Chromatin immunoprecipitation (ChIP)-PCR assay

The ChIP-PCR assay was performed with Simple ChIP Assay Kits (Beyotime Institute of Biotechnology, Hangzhou, ZJ, China, Cell Signaling Technology). Briefly, cells were treated with 37% formaldehyde for 12 min and subjected to ultrasonication on ice. The DNA-protein complexes were immunoprecipitated with anti-RELA or anti-Slug antibody (Abcam, Cambridge, UK). The bound DNA fragments were then isolated by ChIP reactions and subjected to PCR using primers specific to the promoters of CD44 or ESA. The primers used in the ChIP assay are listed in Additional file 4: Supplementary Table S5 [24, 25].

### RIP assay

RNA pulldown assays were performed using a Target RNA Purification Kit (Shanghai Zeheng Biotech, China). All of the following experiments were performed as described previously [26] with small modifications. The probes used are listed in Supplementary Table S6. Cells were crosslinked by formaldehyde, equilibrated in glycine buffer and scraped with lysis buffer. Cell samples were sonicated and then centrifuged. The supernatants were transferred to a 2-ml tube, and 50 μl was saved as the input sample. Cell lysates were incubated with the biotin-tagged-specific probe or control probe for 3 h. The lysate supernatant was incubated with streptavidin beads for 1 h with rotation. The beads-sample mixture was washed. Subsequently, 10% of the beads-sample mixture was subjected to TRIzol extraction. Purified mRNA and miRNAs were detected by qRT-PCR assay using an All-in-One miRNA qRT-PCR Detection Kit (Gene Copoeia, USA).

### In vivo experiment

Female nonobese diabetic/severe combined immunodeficient (NOD/SCID) mice at 6 weeks of age and weighing 18 ± 1 g were ordered from Beijing Weitong Lihua Experimental Animal Technology Co., Ltd., China. All mice were raised in an SPF level animal facility at the Experimental Animal Center, School of Medicine, Southeast University. The animal experiments followed the guidelines of the Animal Research Ethics Board of Southeast University.

BCSCs (2 × 10^5^) were injected into the right inguinal mammary fat pads in each mouse. Twenty-one days after injection, tumors of about 3–5 mm were found in the mice. At this point, twelve tumor-bearing mice were randomly divided into the following three groups of equal numbers (four per group): (1) the BCSC group (phosphate-buffered saline (PBS) locally injected into the BCSC-driven tumor site); (2) the drug group received the following: 30 mg/kg, adriamycin (Ad, purchased from Dalian Meilun Biotech Co., Ltd. of China, twice a week for a total of 3 weeks) and 10 mg/kg cyclophosphamide (Cy, purchased from Dalian Meilun Biotech Co., Ltd. of China, once a week for 3 weeks in 0.1 ml PBS); both were locally injected into the BCSC-formed tumor site; and (3) the group treated with miR-7 agomir, which was synthesized by the Rioribo Company and performed as described previously, whereby 2 nmol miR-7 agomir in 0.1 ml saline buffer was locally injected into the tumor site once every 3 days for a total of 7 times. Tumor development was assessed for up to 42 days. The tumor tissues were subjected to Western blotting, RT**-**qPCR, and immunohistochemistry assays. All experiments were repeated twice [16].

### Statistics

Values of interest are presented as the mean plus or minus standard deviation. Statistical comparisons were performed using Student’s *t* test. A *P* value < 0.05 was considered statistically significant.

## Results

### MiR-7 and BCSC-related molecular expression in breast cancer

**Fig. 1:**
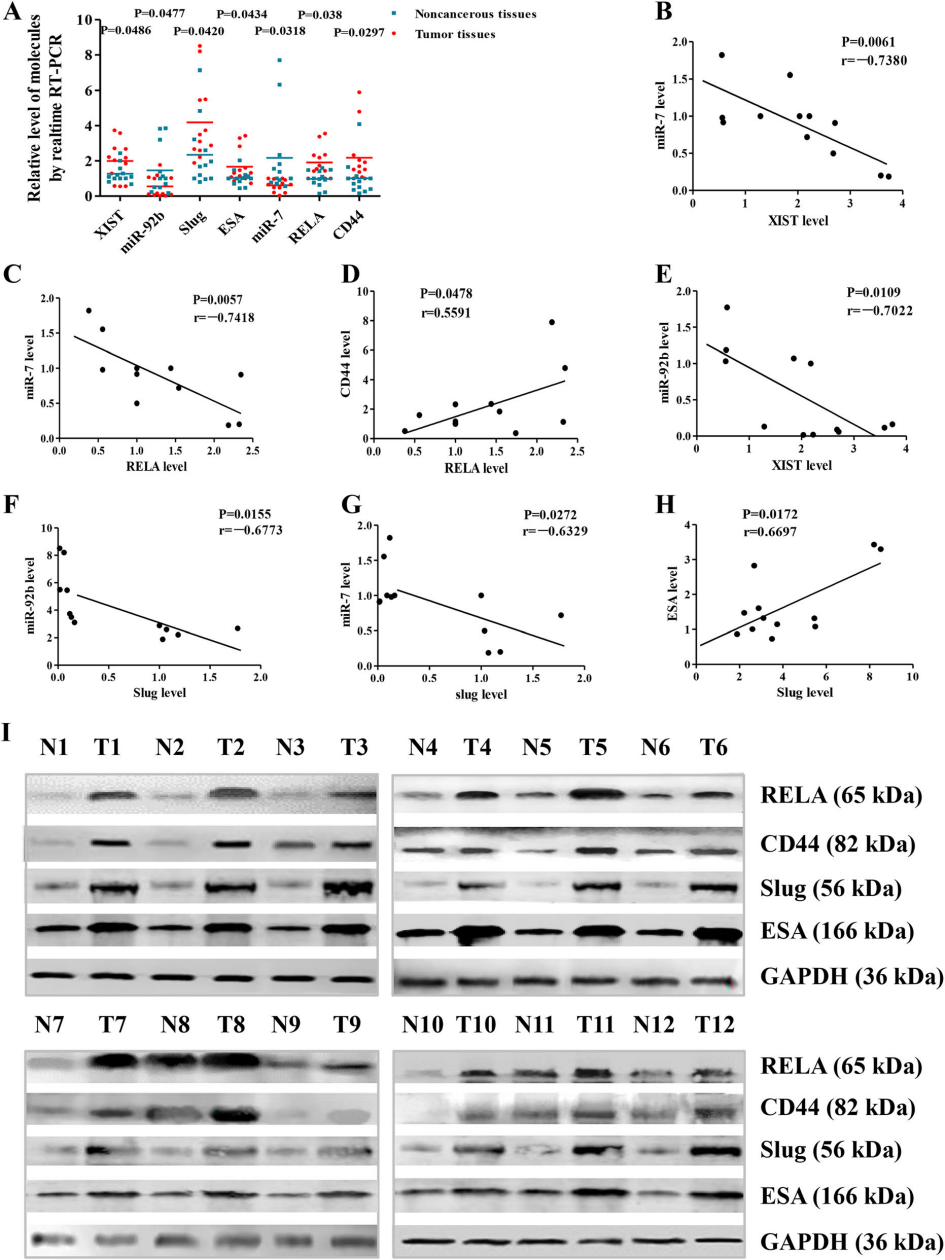
Detection of miR-7 and BCSC-related molecular expression. **a** Expression of miR-7, XIST, miR-92b, RELA, CD44, Slug, and ESA in breast cancer postsurgery samples analyzed by RT-qPCR. **b**–**h** Relative expression levels of miR-7 and XIST, miR-7 and RELA, miR-7 and Slug, miR-92b and XIST, miR-92b and Slug, RELA and CD44, and ESA and Slug in that order. **i** Relative expression levels of RELA, CD44, Slug, and ESA analyzed by Western blotting. All the data represent the mean ± S.D. (*n* = 12). Blue points represent adjacent noncancerous tissues; red points represent tumor tissues. N noncancerous tissues, T tumor tissues

To identify miR-7 and BCSC-related molecular expression levels in breast cancer, we collected 12 postsurgery samples from breast cancer patients and used them in RT-qPCR. The results showed that mostly miR-7 expression was significantly lower in breast cancer tissues than in the adjacent noncancerous tissues (*p =* 0.0318). Based on these results, we further investigated whether the expression of XIST, miR-92b, RELA, CD44, slug, and ESA was related to low miR-7 expression. In contrast to miR-7 expression, the relative expression levels of XIST, RELA, CD44, slug, and ESA were significantly higher in breast cancer tissues than in adjacent noncancerous tissues (*p =* 0.0486, *p =* 0.038, *p =* 0.0297, *p =* 0.0420, and *p =* 0.0434, respectively) (Fig. 1a).

Interestingly, the lower miR-7 was, the higher XIST, RELA, CD44, slug, and ESA were, but the relative expression levels of XIST, RELA, CD44, slug, and ESA changed concurrently (Fig. 1c–e, g, h). Moreover, the expression levels of RELA, CD44, Slug, and ESA were further confirmed by Western blotting analyses (Fig. 1i). In addition, we found that miR-92b expression was similar to that of miR-7 and was decreased in breast cancer tissues compared with the adjacent noncancerous tissues (*p =* 0.0477).

### MiR-7 regulates the expression of BCSC-related molecules in breast cancer cells

**Fig. 2:**
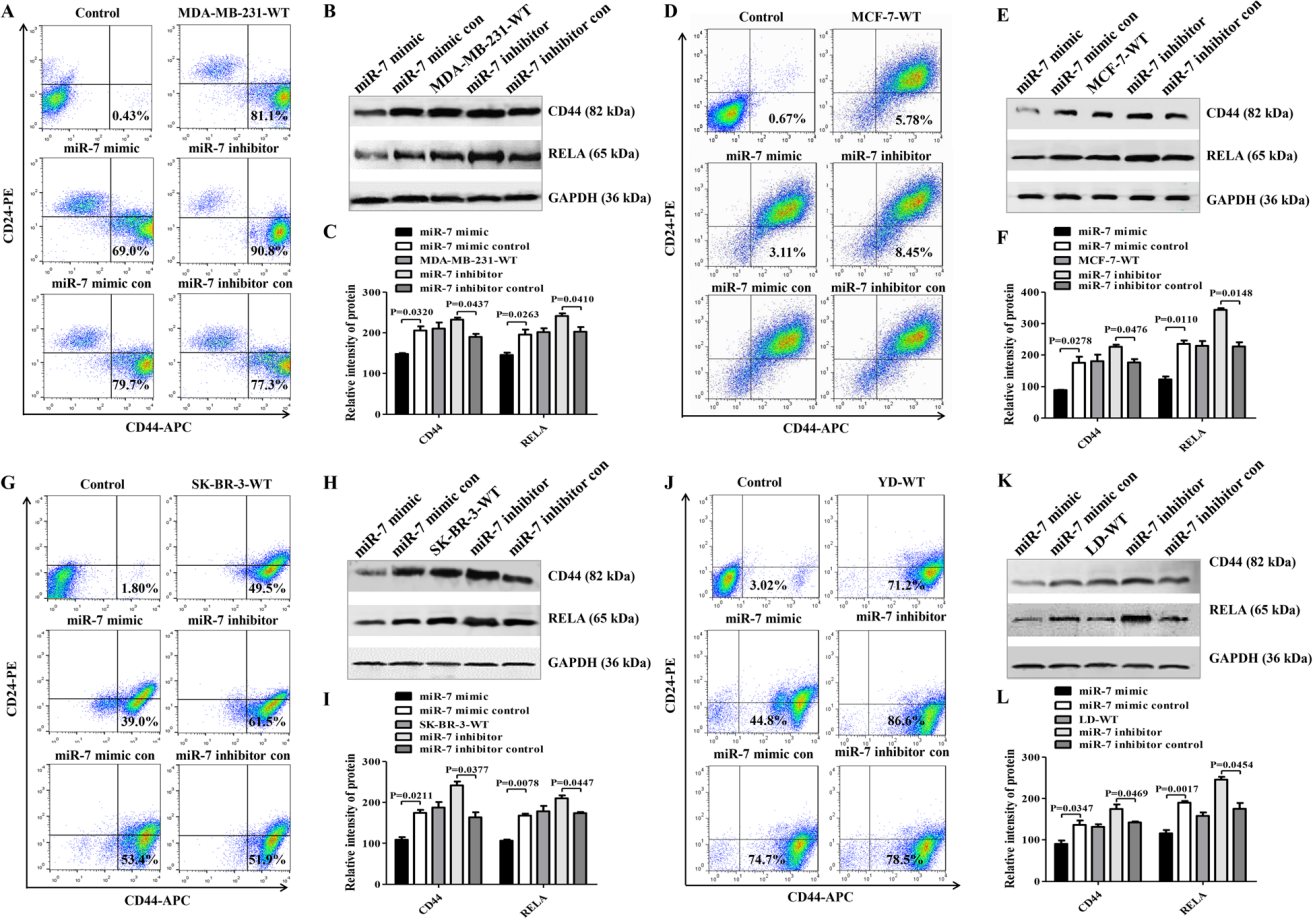
BCSC-related molecular expression regulated by miR-7 mimic. **a**, **d**, **g**, **j** MDA-MB-231, MCF-7, SK-BR-3, and LD cells transfected with miR-7 mimic, respectively. After 48 h of transfection, CD44^+^CD24^−^ or ESA^+^CD44^+^CD24^−^ cell phenotypes were analyzed by FCM. **b**, **e**, **h**, **k** Western blotting analysis of RELA and CD44 expression. **c**, **f**, **i**, **l** Semiquantitative analysis of RELA and CD44 expression

RT-qPCR showed that miR-7 expression in clinical samples was negatively correlated with the expression of XIST, RELA, CD44, slug, and ESA. We further evaluated whether these expression levels would be changed in breast cancer cell lines after miR-7 mimic transfection. Since the CD44 molecule may serve as a phenotypic marker of BCSCs [3], we first assessed CD44 expression by flow cytometry (FCM). It is well known that MDA-MB-231 cells, which are epithelial cell carcinomas from estrogen receptor (ER^−^), progesterone receptor (PR^−^), and epidermal growth factor receptor 2 (HER2^−^) triple-negative breast cancer (TNBC), possess higher invasion and metastasis capability than MCF-7 cells, which are duct cell carcinomas with (ER^+^/PR^+^/HER2^−^) profile [2, 16, 27]. The LD cell line (ER^−^/PR^−^/HER2^+^) was successfully established directly from a human breast cancer postsurgery sample (Figure S1), while the SK-BR-3 cell line exhibits ER^−^/PR^−^/HER2^+^. Interestingly, FCM analysis showed that CD44 expression was concurrently decreased in the four cell lines transiently transfected with miR-7 mimic, but not with miR-7 mimic control; CD44 expression was simultaneously rescued in MDA-MB-231 (Fig. 2a), MCF-7 (Fig. 2d), SK-BR-3 (Fig. 2g), and LD cells (Fig. 2j) after transfection with a miR-7 inhibitor. Similarly, the expression levels of XIST, slug, and ESA were synchronously reduced but miR-92b expression was increased in the miR-7 mimic transiently transfected cells (shown in Additional file 2: Figures S2 a to d).

Since NF-κB subunit RELA function may be implicated in CD44 expression [27], we further analyzed RELA and CD44 expression by RT-qPCR and Western blotting. Consistent with the XIST, Slug, and ESA expression patterns, CD44 and RELA expression levels were also reduced in the miR-7 mimic-transfected cells (Additional file 2: Figures S2 e to g, Fig. 2b, e, h, and k). Furthermore, CD44 expression was concomitantly decreased in RELA-downregulated cells (Additional file 3: Figure S3). It is thus evident that the synthetic miR-7 mimic acts as a regulator to modulate the expression of BCSC-related molecular genes in breast cancer cell lines.

### MiR-7 inhibits CD44 expression by targeting RELA

**Fig. 3:**
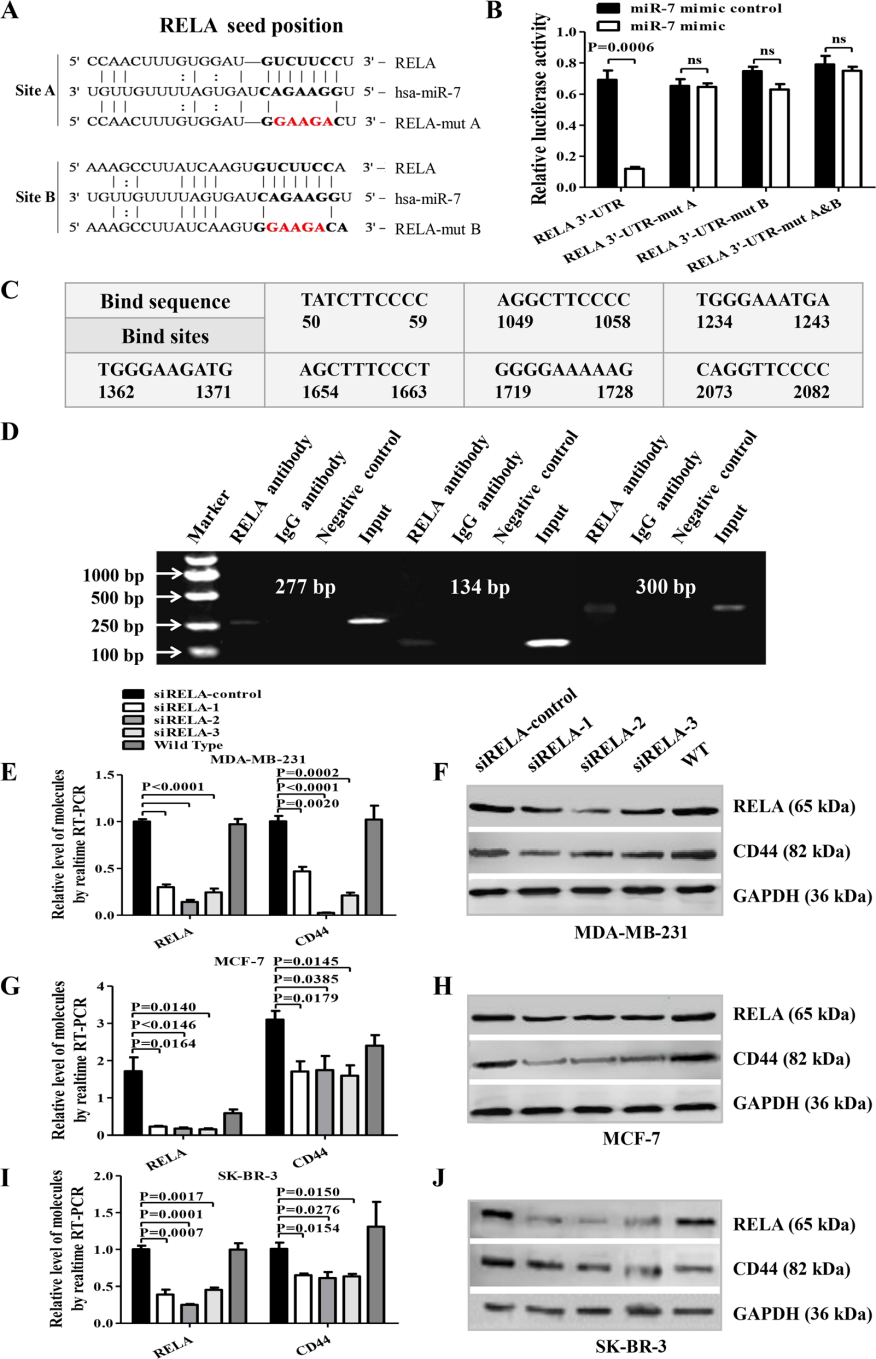
MiR-7 decreases CD44 expression by directly targeting the 3′UTR of RELA. **a** Putative miR-7 wild-type and mutated binding sites in RELA. **b** Luciferase reporter activity. **c** Representation of *CD44* promoter shows six RELA-binding sites **d**. PCR-ChIP assays. In MDA-MB-231 cells, putative RELA-binding sites were identified at regions − 1234 to − 1243, − 1654 to − 1663, and − 2073 to − 2082 in the *CD44* promoter. **e**–**j** The RELA and CD44 transcriptional and translational expression levels following siRELA transfection of MDA-MB-231, MCF-7, and SK-BR-3 cells

To explore the mechanism of miR-7-mediated regulation of RELA and CD44 expression, a dual-luciferase reporter assay was first conducted in MDA-MB-231 cells. By TargetScan and miRcode algorithm prediction, we found that RELA was one of the miR-7 candidate genes and the RELA 3′-UTR contained two miR-7 binding sites (Fig. 3a). The following reporter assay was performed as previously described [6, 16]. The results showed the miR-7 mimic reduced the relative luciferase activity of the wild-type vector, which contained the putative miR-7 binding sites in the RELA 3′-UTR, but not the relative luciferase activity of the mutant A or B vector or the A plus B vector (Fig. 3b). Next, we tested whether RELA had enhanced occupancy in the promoter region of its target gene *CD44* and conducted a ChIP assay. Based on the JASPAR database prediction, we found that there were seven putative RELA-binding sites in the *CD44* promoter (Fig. 3c). ChIP-PCR results indicated that RELA was directly bound to the − 1234 to − 1243, − 1654 to − 1663, and − 2073 to − 2082 regions in the *CD44* promoter in MDA-MB-231 cells (Fig. 3d). To further confirm these findings, we investigated whether silencing RELA could decrease CD44 expression in MDA-MB-231, MCF-7, and SK-BR-3 cells. As shown in Fig. 3e–j, the CD44 transcriptional and translational expression levels were significantly decreased after transfection with siRELA recombinants in comparison with the control cells.

### MiR-7 directly targets XIST and slug to decrease ESA but increases miR-92b expression

**Fig. 4:**
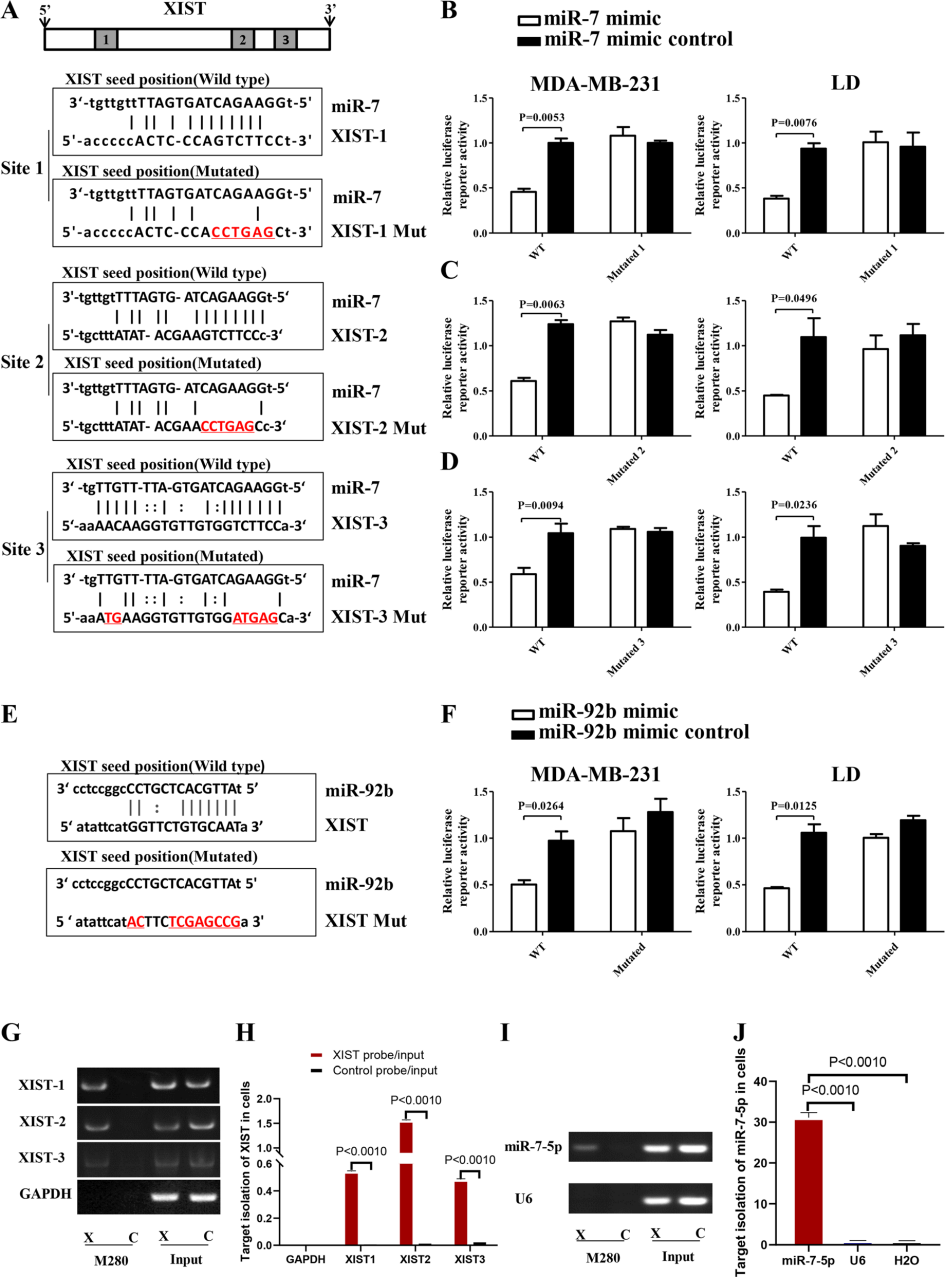
MiR-7/miR-92b specifically bind to XIST in cells. **a** Putative miR-7 wild-type and mutated binding sites in XIST. **b**–**d** Luciferase reporter activity in MDA-MB-231 and LD cells. **e** Putative miR-92b binding and mutated sites in XIST. **f** Luciferase reporter activity in MDA-MB-231 and LD cells. **g** Specific isolation of XIST in MDA-MB-231 cells by RIP assay. **h** Quantitative analysis of XIST pulldown efficiency in cells. **i** RT**-**qPCR analysis of specific isolation of miR-7-5p from the precipitation of the XIST complex in MDA-MB-231 cells. **j** Quantitative analysis of miR-7-5p pulldown efficiency in the precipitation of the XIST complex in cells

To explore the effects of miR-7 on XIST and the miR-92b/Slug/ESA axis, we found that XIST contained three predicted binding sites for miR-7 (Fig. 4a) and one for miR-92b (Fig. 4e) based on TargetScan and miRcode algorithm prediction. The results indicated that the miR-7 or miR-92b mimic significantly decreased the relative luciferase activity of the wild-type vector compared with the control (Fig. 4b–f), suggesting that the inhibition of XIST expression was regulated by miR-7 and miR-92b. Next, we found that there were no sites for miR-7 in the ESA 3′UTR, but the slug mRNA contained one, as shown in Fig. 4g. The result in Fig. 4h shows that miR-7 reduced the relative luciferase activity of the wild-type vector but not the mutant vectors, indicating the inhibition of slug expression. Additionally, we further used biotin-tagged XIST antisense oligonucleotides (XIST probe) and performed RIP assay to pull down the XIST complex by beads and then identified the pulldown of miR-7-5p from the XIST complex precipitate to confirm miR-7 targeting of XIST in MDA-MB-231 cells [26]. Figure 4i demonstrated the specific isolation of XIST from the XIST probe and control probe (input) in MDA-MB-231 cells. RT-PCR analysis showed that XIST was efficient in cells, especially in isolation of XIST2, as shown in Fig. 4j. Specific isolation of miR-7-5p from the XIST probe and control probe (input) in cells is shown in Fig. 4k. Figure 4l represents the RT-PCR analysis of miR-7-5p isolation efficiency in cells. These data strongly demonstrated that, in addition to the dual-luciferase reporter assay, the RIP results further provided evidence that miR-7 could actually bind to XIST in MDA-MB-231cells.

**Fig. 5:**
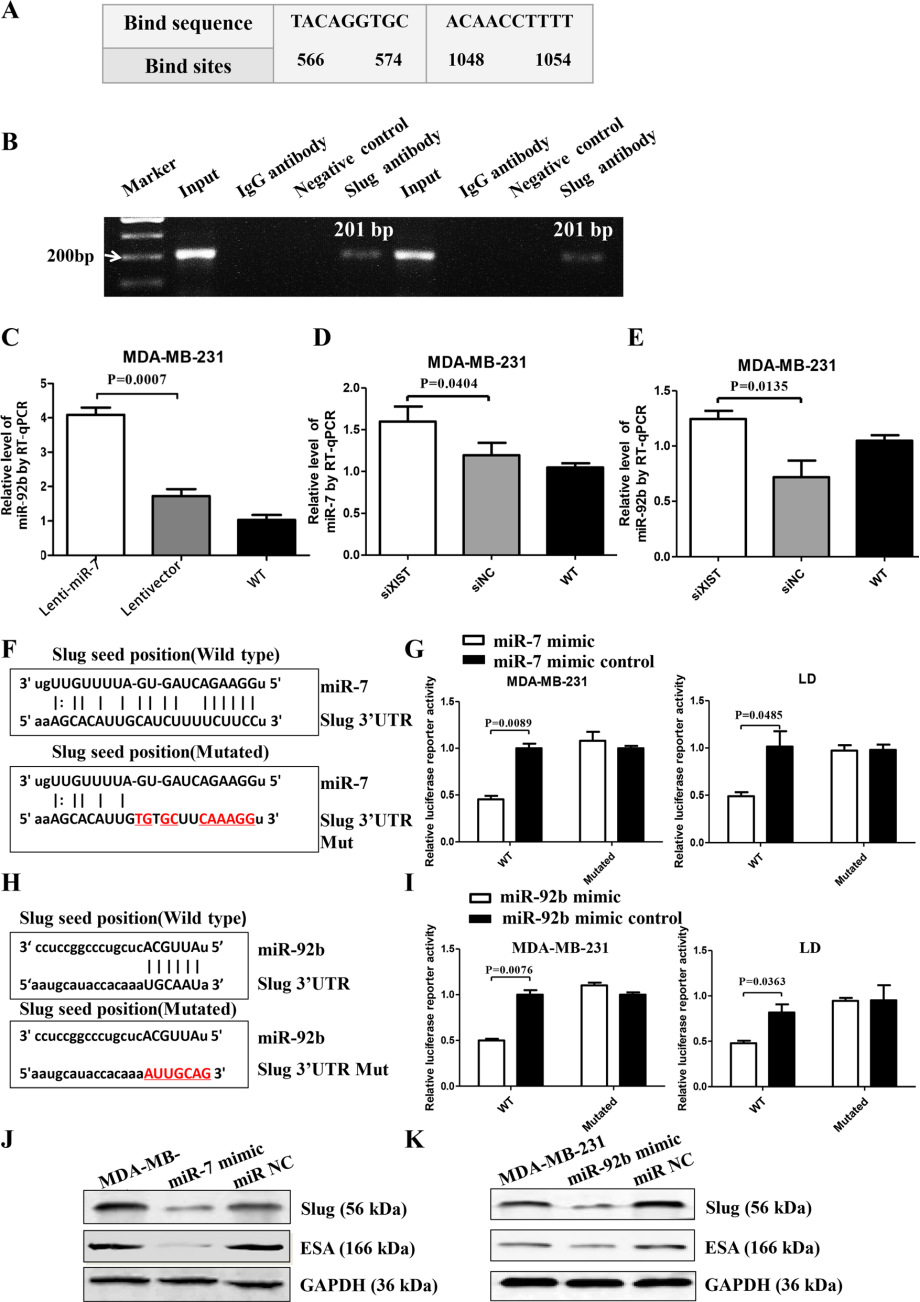
MiR-7/miR-92b targets slug to decrease ESA expression. **a** Representation of the human *ESA* promoter showing slug binding at two sites. **b** PCR-ChIP assay results. **c** RT**-**qPCR analysis of miR-92b expression in Lenti-miR-7-infected cells. **d** RT**-**qPCR analysis of miR-7 expression after silencing XIST. **e** RT**-**qPCR analysis of miR-92b expression after silencing XIST. **f** Putative miR-7 wild-type and mutated binding sites in slug. **g** Luciferase reporter activity in MDA-MB-231 and LD cells. **h** Putative miR-92b wild-type and mutated binding sites in Slug. **i** Luciferase reporter activity in MDA-MB-231 and LD cells. **j** Western blot analysis of Slug and ESA expression regulated by miR-7. **k** Western blot analysis of Slug and ESA expression regulated by miR-92b

Furthermore, we conducted ChIP-PCR in MDA-MB-231 cells to determine whether slug would directly increase ESA transcription by binding to the *ESA* promoter region. Figure 5a shows two putative Slug-binding sites in the *ESA* promoter. ChIP-PCR results indicated that Slug was bound to PCR-amplified fragments at two DNA binding sites (Fig. 5b).

Interestingly, RT-qPCR results also showed that miR-92b expression was increased in Lenti-miR-7-infected MDA-MB-231 cells (Fig. 5c), which may be due to inhibition of XIST. To verify this, we silenced XIST by siRNA and found that knockdown of XIST enhanced miR-7 and miR-92b expression, as shown in Fig. 5d, e. More interestingly, there was one binding site for miR-92b in the Slug 3′UTR (Fig. 5f). Figure 5j shows that miR-92b decreased the relative luciferase activity of the wild-type Slug 3′UTR vector due to the inhibition of slug expression in MDA-MB-231 and LD cells. Moreover, we validated the RT-qPCR results by Western blotting and found that miR-7 and miR-92b respectively inhibited the expression of slug and ESA in MDA-MB-231 cells following transfection of miR-7 or miR-92b mimic (Fig. 5h, i). Overall, although Slug is a strong transcription factor and directly bound to regions in the *ESA* promoter to induce ESA expression, miR-7 and miR-92b could directly downregulate Slug expression, resulting in reduced ESA expression.

### MiR-7 reduces BCSC-driven tumor growth by decreasing the BCSC subset

**Fig. 6:**
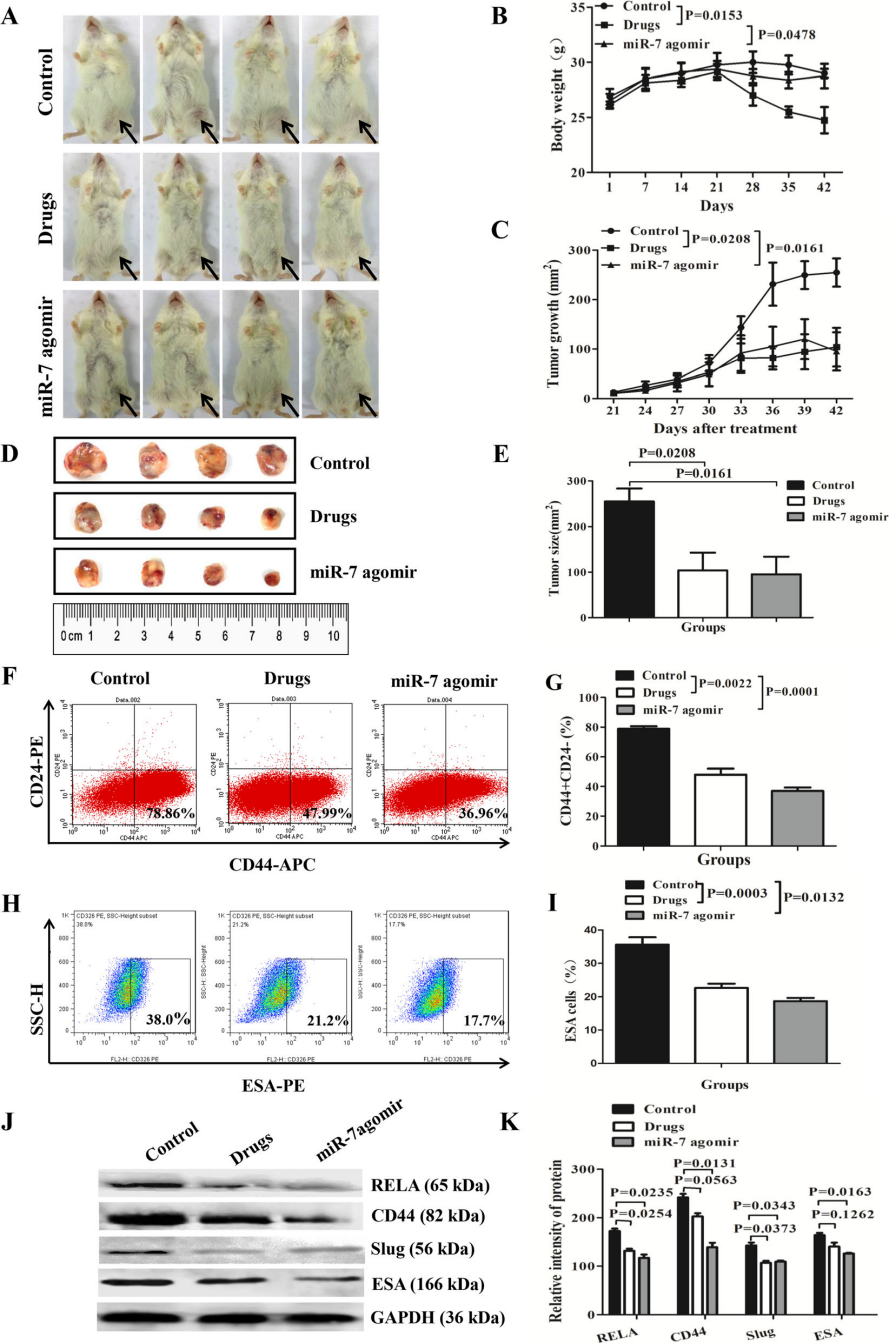
MiR-7 agomir reduces BCSC-driven xenograft growth. **a** Representative images show the BCSC xenograft growth in NOD/SCID mice 21 days after treatment with Ad+Cy, miR-7 agomir, or PBS. **b** Mouse weight changes. **c** Tumor dynamic growth plot. **d** Tumor sizes 21 days after treatment. **e** Quantification of tumor sizes. **f**–**i** FCM analysis of CD44^+^CD24^−^ and CD44^+^CD24^−^ESA^+^ BCSC percentages. **j**, **k** Western blotting analysis of CD44, RELA, Slug, and ESA expression levels in tumor tissues

Furthermore, we evaluated the potential role of miR-7 agomir in suppressing BCSC growth in vivo. To this end, we established a BCSC**-**driven xenograft model by locally injecting of 2 × 10^5^ BCSCs, which were isolated from MDA-MB-231 cells, into the right inguinal mammary fat pads of NOD/SCID mice. All mice generated tumor xenografts 17 days after injection. The tumor-bearing mice were treated with miR-7 agomir, Ad plus Cy, or PBS control 4 days later. Figure 6a contains photographs of breast cancer-bearing mice 21 days after treatment. The mouse weight changes and the dynamic tumor growth were monitored (Fig. 6b, c). As shown in Fig. 6b, we found that weight loss was approximately 5% on day 7 after initiation of miR-7 agomir treatment (from day 21 to day 28), and it mostly maintained mouse weight similar to that of control mice 21 days after treatment (from day 21 to day 42). This suggested that local injection of the miR-7 agomir caused no severe side effects. However, in response to the combined Ad plus Cy treatment, the mice showed serious weight loss, suggesting severe side effects in the drug-treated mice.

Figure 6d shows photographs of the tumors 21 days after treatment. The quantification analysis of tumor sizes is shown in Fig. 6e, in which the tumor sizes were slightly smaller in mice treated with miR-7 agomir than mice treated with Ad plus Cy, but the difference was not statistically significant (*p* > 0.05). Furthermore, the tumor sizes in the two groups of mice were much smaller than those in the mice treated with PBS control (*p* < 0.0208 or *p* < 0.0161). The counts of CD44^+^CD24^−^ BCSCs (Fig. 6f) and CD44^+^CD24^−^CD44^+^ BCSCs (Fig. 6h) were also significantly decreased in the tumor tissues of mice treated with the miR-7 agomir and in the Ad plus Cy group (Fig. 6g–i). Western blotting results showed that the expression levels of REAL, CD44, Slug, and ESA were concurrently reduced (Fig. 6j–k).

## Discussion

Crucial concerns for the BCSC subset are nearly related with the chemoresistance and recurrence of breast cancer; we explored the mechanisms of decreasing the BCSC subset regulated by miR-7. Our findings in the present study revealed that miR-7 and miR-92b expression levels were markedly decreased in breast cancer surgery samples in contrast to the high expression of lncRNA XIST, RELA, CD44, slug, and ESA, suggesting a negative correlation between miR-7/miR-92b and the expression of BCSC stemness-related molecules in vivo. In Fig. 2 and Figure S2 a to d, we found that the expression levels of XIST, RELA, CD44, Slug, and ESA were remarkably decreased in the miR-7 mimic transiently transfected cells, especially in the case of XIST, which may regulate the stemness characteristics and tumorigenicity of some types of cancer cells, resulting in cancer progression [28–33]. To verify these claims, we attempted to identify whether XIST expression was regulated by miR-7/miR-92b. The results indicated that miR-7/miR-92b could significantly inhibit the reporter activity of XIST, whereas knockdown of XIST led to high miR-7/miR-92b expression, which suggested the reciprocal regulation between miR-7 and XIST in MDA-MB-231 and LD cells.

To further confirm that miR-7 directly targets XIST in breast cancer, we performed a RIP assay. The results in Fig. 4 g to j showed that miR-7 could directly bind to XIST in MDA-MB-231 cells, which fully demonstrated that miR-7 could inhibit XIST expression.

It is known that RELA is not only a member of the NF-κB family and participates in many biological processes but is also involved in the NF-κB signaling pathway, which correlates with BCSC proliferation activity [28]. Thus, we evaluated whether RELA could directly regulate CD44 expression. As expected, ChIP-PCR results showed that RELA had increased occupancy in the promoter region of *CD44*, and this was further confirmed by knockdown of RELA, which resulted in reduced CD44 expression. Next, we observed that miR-7 could directly regulate RELA expression, as confirmed by the luciferase reporter assay. Since CD44 is tightly associated with the BCSC phenotype [10, 16, 34], we hypothesized that miR-7 decreased the BCSC subset mainly by targeting RELA to inhibit CD44 expression. Our findings agreed with a recent report by Cantley et al., which reported a targeted relationship between miR-7 and the NF-κB subunit RELA and miR-7 inhibition of RELA expression could impact the proliferation, invasion, and apoptosis of non-small cell lung cancer cells. However, the report did not involve the CSC subset [35].

In the present study, we also observed low miR-92b and high XIST expression concurrently in breast cancer tissues and cell lines. These findings were in agreement with several studies on XIST and miR-92b expression in tumors [13–15, 36–38].

In addition, we found that miR-7 negatively regulated XIST expression but positively regulated miR-92b expression and that high miR-92b expression enhanced the inhibition of transcription factor Slug activity, resulting in decreased ESA expression. The miR-7 mimic not only decreased ESA expression by indirectly increasing miR-92b expression to inhibit slug activity but also directly downregulated Slug to reduce ESA expression. These epigenetic regulatory effects finally led to a decrease in the BCSC subset percentage in breast cancer.

It is possible that XIST serves as a competing endogenous RNA to bind miR-7 to inhibit its function. Since while XIST was knocked down, the expression of miR-7 was clearly increased. Similarly, miR-92b exhibited an inverse relationship with XIST gene expression. Consequently, the results from the in vitro experiments demonstrated that the miR-7 mimic could exert multiple reciprocal regulatory actions to inhibit the expression of XIST, RELA, Slug, CD44, and ESA but increase miR-92b expression.

In vivo animal experiments demonstrated that the local treatment of the BCSC-driven xenograft with the miR-7 agomir had the same effects as applying the combined treatment of Ad plus Cy in a mouse model, which resulted in a reduction in tumor growth and improvement of tumor-bearing mouse survival as well as decrease in BCSC phenotypes (CD44, ESA). A combination of Ad plus Cy treatment of breast cancer is well known as one of the conventional therapies in the past few decades [39, 40]. Our results suggested a potential value of miR-7 agomir as a local treatment reagent in the treatment of breast cancer.

## Conclusions

**Fig. 7:**
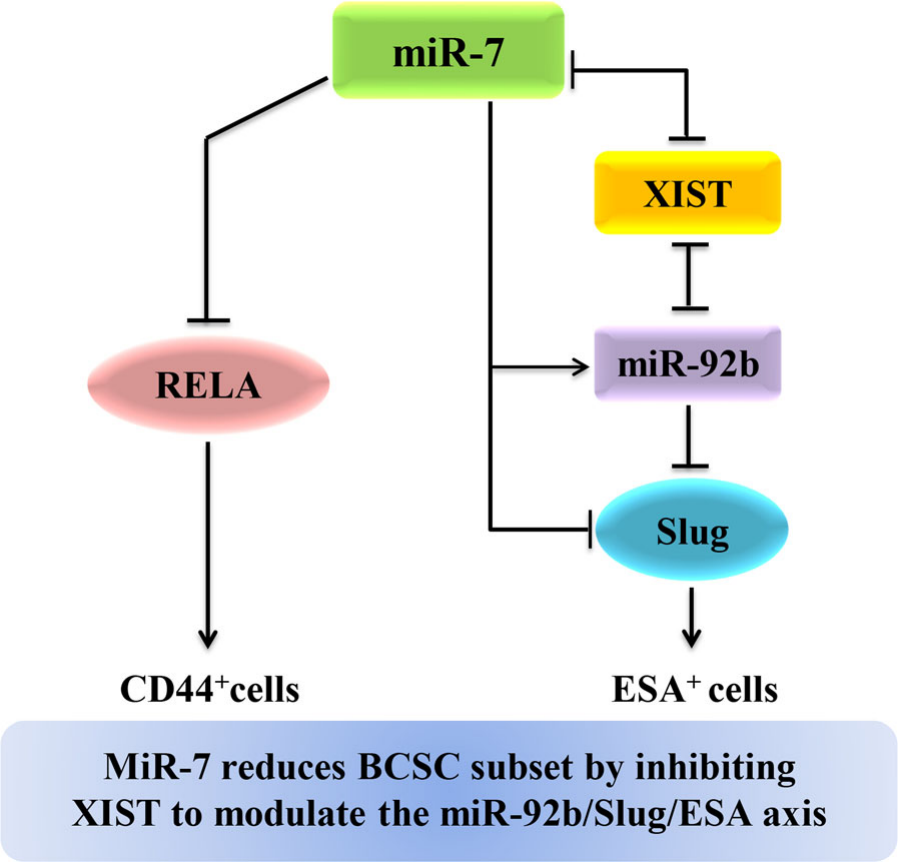
MiR-7 decreases the BCSC subset by inhibiting XIST to modulate the miR-92b/Slug/ESA axis. Notes: XIST long noncoding RNA X-inactive-specific transcript, ESA epithelium-specific antigen. → Promoting; ⊥ inhibiting;  reciprocal inhibiting

In this study, we first demonstrate that miR-7 not only effectively targeted XIST to regulate the miR-92b/Slug/ESA axis but also decreased RELA and CD44 expression to reduce the BCSC subset (as shown in Fig. 7). Significantly, the findings are of potential clinical importance in understanding the multiple regulatory roles of miR-7. The overexpression of miR-7 and/or the knockdown of XIST may contribute to a significant approach to the treatment of breast cancer.

## Supplementary information

**Additional file 1: Figure S1.** The LD cell line was established and identified from human breast cancer sample.

**Additional file 2: Figure S2.** Expression of XIST, Slug, ESA, miR-92b, RELA, and CD44 determined by RT-qPCR.

**Additional file 3: Figure S3.** FCM analysis of the CD44 phenotype in breast cancer cells transfected with siRELA recombinants.

**Additional file 4: Table S1.** Clinical specimen data. **Table S2.** The primers used for q-PCR. **Table S3.** The microRNA mimic and siRNA sequences. **Table S4.** The primers used in the dual-luciferase reporter assay. **Table S5.** The primers for ChIP-PCR.

## Data Availability

All data generated or analyzed during this study are included in this published article.

## Abbreviations

BCSCs: Breast cancer cells
ChIP: Chromatin immunoprecipitation
ER: Estrogen receptor
HER2: Epidermal growth factor receptor 2
miR-7: Noncoding microRNA
NOD/SCID: Nonobese diabetic/severe combined immunodeficient
PR: Progesterone receptor
XIST: X-inactive-specific transcript
