# Supplementary figures and images for: MiR-7 reduces the BCSC subset by inhibiting XIST to modulate the miR-92b/Slug/ESA axis and inhibit tumor growth
Source: Breast Cancer Res. 2020 Mar 6;22:26. doi: 10.1186/s13058-020-01264-z (PMC7060548; doi:10.1186/s13058-020-01264-z)

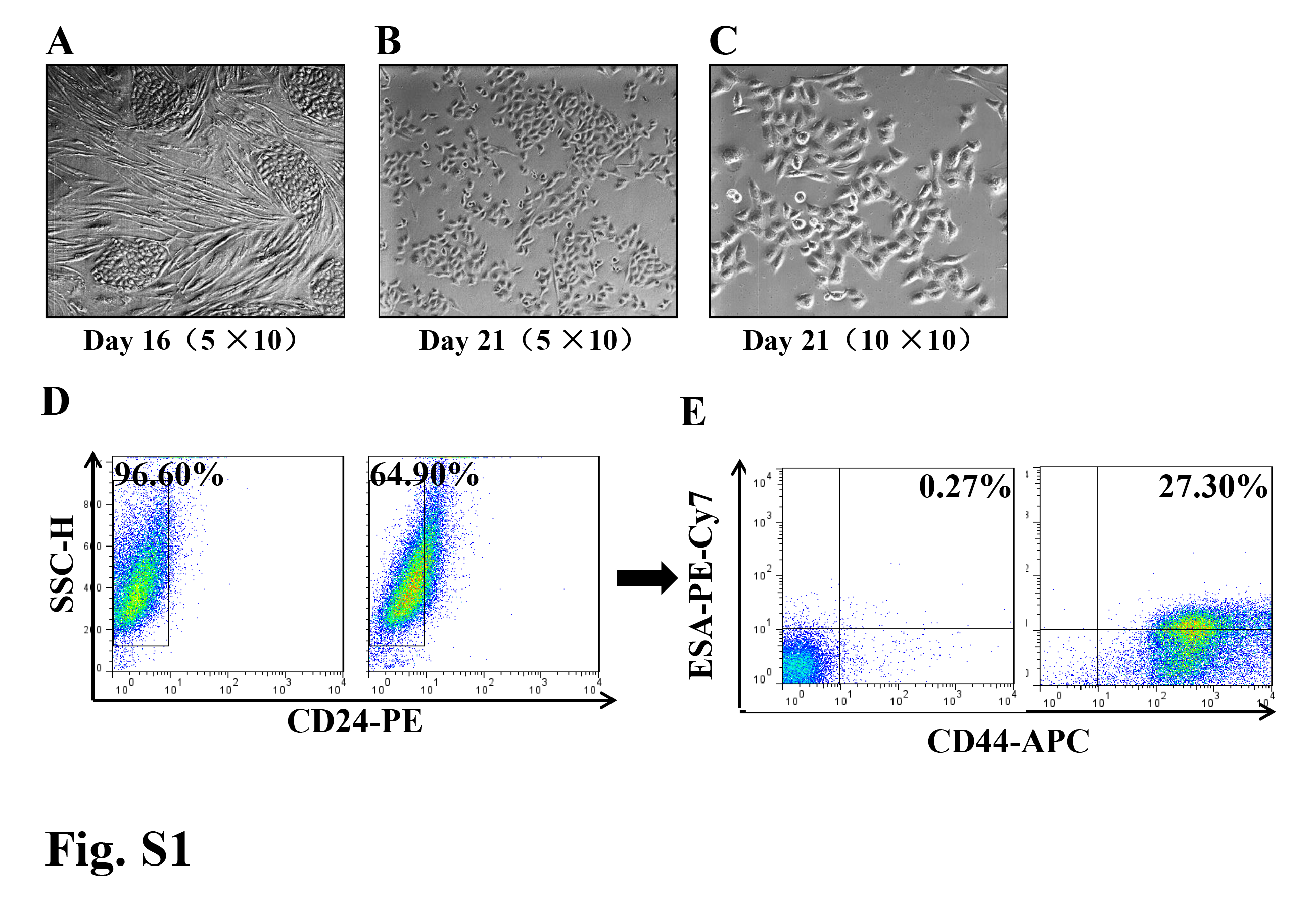

Supplement: Supplementary file 1 — Additional file 1: Figure S1. The LD cell line was established and identified from human breast cancer sample. [file 13058_2020_1264_MOESM1_ESM.tif]

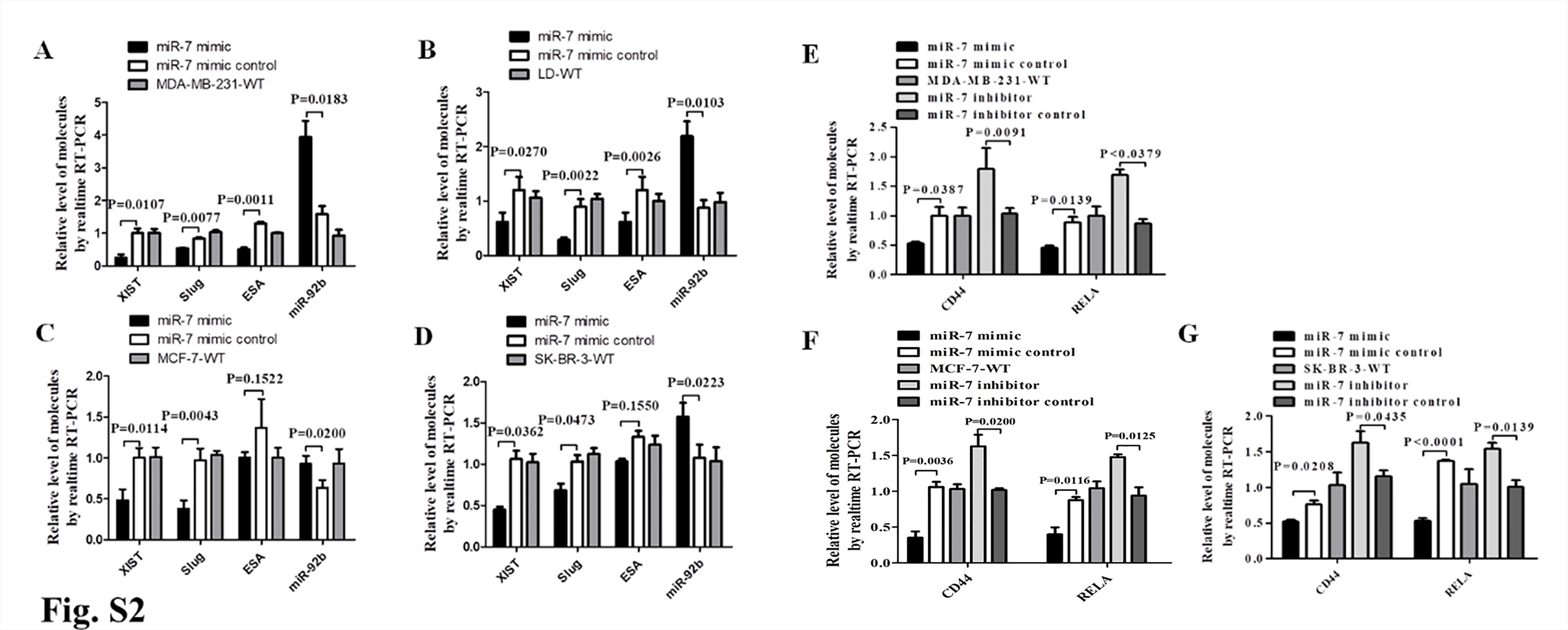

Supplement: Supplementary file 2 — Additional file 2: Figure S2. Expression of XIST, Slug, ESA, miR-92b, RELA, and CD44 determined by RT-qPCR. [file 13058_2020_1264_MOESM2_ESM.tif]

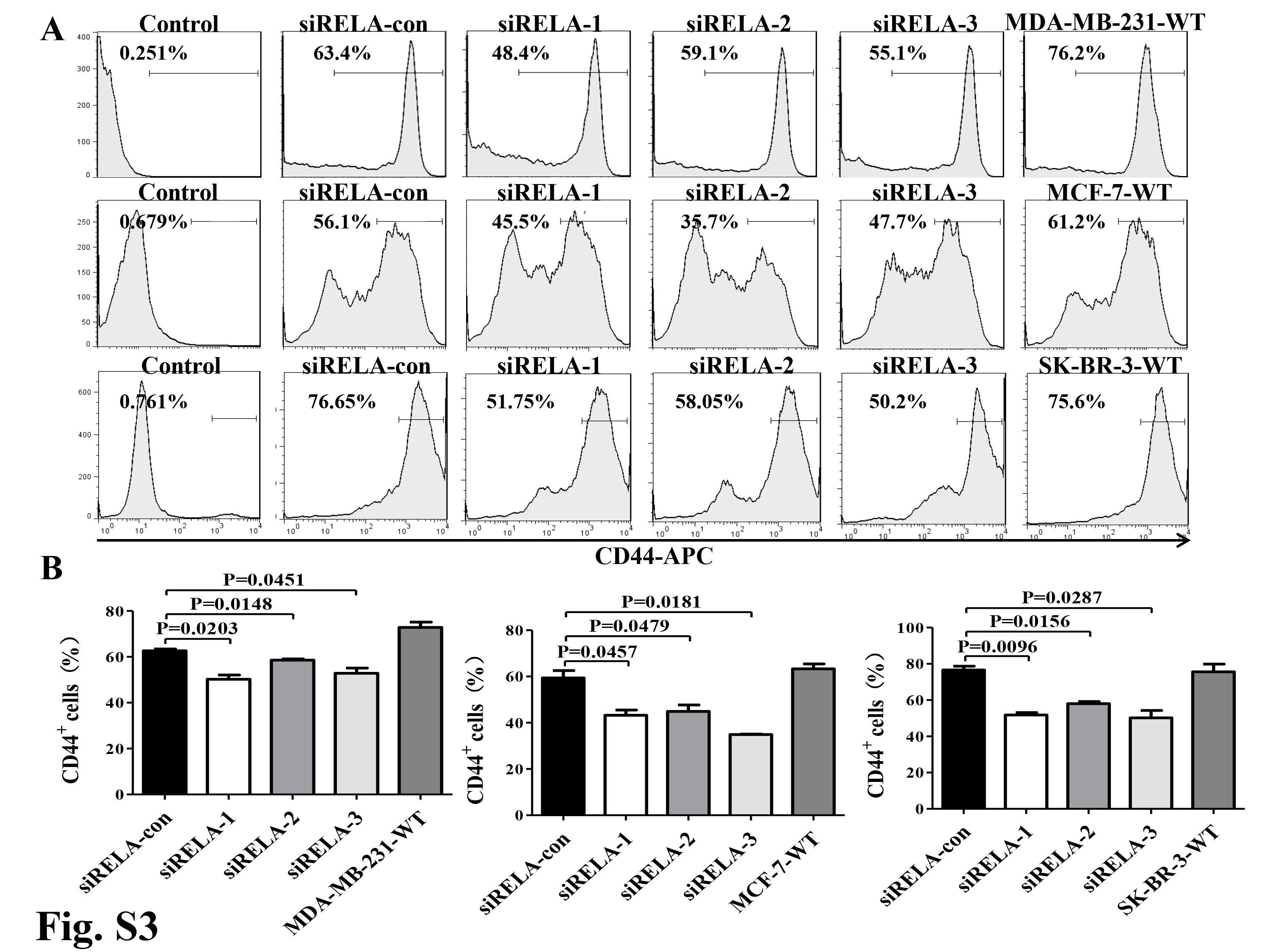

Supplement: Supplementary file 3 — Additional file 3: Figure S3. FCM analysis of the CD44 phenotype in breast cancer cells transfected with siRELA recombinants. [file 13058_2020_1264_MOESM3_ESM.tif]
