# Supplementary material for: MiR-7 reduces the BCSC subset by inhibiting XIST to modulate the miR-92b/Slug/ESA axis and inhibit tumor growth
Source: Breast Cancer Res. 2020 Mar 6;22:26. doi: 10.1186/s13058-020-01264-z (PMC7060548; doi:10.1186/s13058-020-01264-z)
Supplement: Supplementary file 4 — Additional file 4: Table S1. Clinical specimen data. Table S2. The primers used for q-PCR. Table S3. The microRNA mimic and siRNA sequences. Table S4. The primers used in the dual-luciferase reporter assay. Table S5. The primers for ChIP-PCR. [file 13058_2020_1264_MOESM4_ESM.doc]

**Supplementary Tables**

**Supplementary Table S2. The primers used for q-PCR**

| Gene name | Forward primers(5’-3’) | Reverse primers(5’-3’) |
| --- | --- | --- |
| MiR-7 | ACACTCCAGCTGGGTGGAAGACTAGTGATTT | CTCAACTGGTGTCGTGGAGTCGGCAATTCAGTTGAGACAACAAA |
| RELA | TGCTTGGCAACAGCACAGA | AGCTGCTGAAACTCGGAGTTGT |
| CD44 | ACACGAAGGAAAGCAGGACC | TTTGCTCCACCTTCTTGACTC |
| MiR-92-b | ACACTCCAGCTGGGTATTGCACTCGTCCCG | TGGTGTCGTGGAGTCG |
| XIST | CGGGTCTCTTCAAGGACATTTAGCC | GCACCAATACAGAGGAATGGAGGG |
| ESA | GTGCTGGTGTGTGAACACTG | GAAGTGCAGTCCGCAAACTT |
| Slug | TGCGATGCCCAGTCTAGAAA | TTCTCCCCCGTGTGAGTTC |
| SNAI1 | TCCACAAGCACCAAGAGTCCG | CAGGCAGAGGACACAGAACCAG |
| TGF-beta1 | ATGGAGAGAGGACTGCGGAT | CAGTAGTGTTCCCCACTGGTC |
| KLF4 | TCTCTTCGTGCACCCACTTG | GGCATGAGCTCTTGGTAATGG |
| Dkk-1 | GACTGTGCCTCAGGATTGTGT | CAGATCTTGGACCAGAAGTGTCT |
| GAPDH | GAAGGTGAAGGTCGGAGTCA | TTGAGGTCAATGAAGGGGTC |
| β-catenin | TACCTCCCAAGTCCTGTATGAG | TGAGCAGCATCAAACTGTGTAG |
| U6 | CTCGCTTCGGCAGCACA | AACGCTTCACGAATTTGCGT |

**Supplementary Table S3. The microRNA mimic and siRNA sequences**

| Gene name | Effective sequence (5’-3’) | Scramble sequence as negative control |
| --- | --- | --- |
| has-miR-7-5p mimic | UGGAAGACUAGUGAUUUUGUUGUU |  |
| has-miR-92b-3p mimic | UAUUGCACUCGUCCCGGCCUCC |  |
| RELA-1 | GGACATATGAGACCTTCAA | CUUACGCUGAGUACUUCGATT（sense）  UCGAAGUACUCAGCGUAAGTT（antisense） |
| RELA-2 | GCCCTATCCCTTTACGTCA |
| RELA-3 | CCCTGAGCACCATCAACTA |
| si-h-LncRNA XIST | GTATCCTATTTGCACGCTA |  |
| si-h-slug | CAATAAGACCTATTCAACT |  |

**Supplementary Table S4. The primers used in dual-luciferase r**eporter assay.

| Gene name | Forward primers(5’-3’) | Reverse primers(5’-3’) |
| --- | --- | --- |
| RELA 3’UTR | CCGCTCGAGGGTGACGCCTGCCCTCCCCAGAGC | ATAAGAATGCGGCCGCCTAGCCAGCTTGGCAACAGATTTA |
| RELA 3’UTR  Mutant-1 | Ggtgacgcctgccctccccagagcactgggttgcaggggattgaagccctccaaaagcacttacggattctggtggggtgtgttccaactgcccccaactttgtggatggaagacttggaggggggagccatattttattcttttattgtcagtatctgtatctctctctctttttggaggtgcttaagcagaagcattaacttctctggaaaggggggagctggggaaactcaaacttttcccctgtcctgatggtcagctcccttctctgtagggaactctggggtcccccatccccatcctccagcttctggtactctcctagagacagaagcaggctggaggtaaggcctttgagcccacaaagccttatcaagtggaagacatcatggattcattacagcttaatcaaaataacgccccagataccagcccctgtatggcactggcattgtccctgtgcctaacaccagcgtttgaggggctggccttcctgccctacagaggtctctgccggctctttccttgctcaaccatggctgaaggaaaccagtgcaacagcactggctctctccaggatccagaaggggtttggtctgggacttccttgctctccctcttctcaagtgccttaatagtagggtaagttgttaagagtgggggagagcaggctggcagctctccagtcaggaggcatagtttttactgaacaatcaaagcacttggactcttgctctttctactctgaactaataaatctgttgccaagctggctag | |
| RELA 3’UTR  Mutant-2 | ggtgacgcctgccctccccagagcactgggttgcaggggattgaagccctccaaaagcacttacggattctggtggggtgtgttccaactgcccccaactttgtggatggaagacttggaggggggagccatattttattcttttattgtcagtatctgtatctctctctctttttggaggtgcttaagcagaagcattaacttctctggaaaggggggagctggggaaactcaaacttttcccctgtcctgatggtcagctcccttctctgtagggaactctggggtcccccatccccatcctccagcttctggtactctcctagagacagaagcaggctggaggtaaggcctttgagcccacaaagccttatcaagtggaagacatcatggattcattacagcttaatcaaaataacgccccagataccagcccctgtatggcactggcattgtccctgtgcctaacaccagcgtttgaggggctggccttcctgccctacagaggtctctgccggctctttccttgctcaaccatggctgaaggaaaccagtgcaacagcactggctctctccaggatccagaaggggtttggtctgggacttccttgctctccctcttctcaagtgccttaatagtagggtaagttgttaagagtgggggagagcaggctggcagctctccagtcaggaggcatagtttttactgaacaatcaaagcacttggactcttgctctttctactctgaactaataaatctgttgccaagctggctag | |
| RELA 3’UTR  Mutant-3 | ggtgacgcctgccctccccagagcactgggttgcaggggattgaagccctccaaaagcacttacggattctggtggggtgtgttccaactgcccccaactttgtggatggaagacttggaggggggagccatattttattcttttattgtcagtatctgtatctctctctctttttggaggtgcttaagcagaagcattaacttctctggaaaggggggagctggggaaactcaaacttttcccctgtcctgatggtcagctcccttctctgtagggaactctggggtcccccatccccatcctccagcttctggtactctcctagagacagaagcaggctggaggtaaggcctttgagcccacaaagccttatcaagtggaagacatcatggattcattacagcttaatcaaaataacgccccagataccagcccctgtatggcactggcattgtccctgtgcctaacaccagcgtttgaggggctggccttcctgccctacagaggtctctgccggctctttccttgctcaaccatggctgaaggaaaccagtgcaacagcactggctctctccaggatccagaaggggtttggtctgggacttccttgctctccctcttctcaagtgccttaatagtagggtaagttgttaagagtgggggagagcaggctggcagctctccagtcaggaggcatagtttttactgaacaatcaaagcacttggactcttgctctttctactctgaactaataaatctgttgccaagctggctag | |
| miR-7-XIST1 sequence | CCGCTCGAGTGTGCATAACCACTTTGCCCATTCCATCTGAATAAGGTCCTACTCTCAGACCCCTTTTGCAGTACAGCAGGGGTGCTGATCACCAAGGCCCCTTTTCTTGGCCTGTTATGTGCGTGATTATATTTGTCTGGGTTCCTGTGTATTAGACAAGGAAGCCTTCCCCCCGCCCCCACCCCCACTCCCAGTCTTCCTTTCCCTTCCAGCAGGGAGTGCCCCCTCCATAAGATCATTACATTTGGACAATCAAGGTGCACAATTATAAGTGACCACAGCCATGCACCTTGGACATTGCGGCCGCATTC | |
| miR-7-XIST1-Mut sequence | CCGCTCGAGTGTGCATAACCACTTTGCCCATTCCATCTGAATAAGGTCCTACTCTCAGACCCCTTTTGCAGTACAGCAGGGGTGCTGATCACCAAGGCCCCTTTTCTTGGCCTGTTATGTGCGTGATTATATTTGTCTGGGTTCCTGTGTATTAGACAAGGAAGCCTTCCCCCCGCCCCCACCCCCACTCCCACCTGAGCTTTCCCTTCCAGCAGGGAGTGCCCCCTCCATAAGATCATTACATTTGGACAATCAAGGTGCACAATTATAAGTGACCACAGCCATGCACCTTGGACATTGCGGCCGCATTC | |
| miR-7-XIST2 sequence | CCGCTCGAGTAGGCACAATGGCAGTTATCAATGGTTTTCTCCCTCCCTGACCTTGTTAAGCAAGCGCCCCACCCCACCCTTAGTTTCCCATGGCATAATAAAGTATAAGCATTGGAGTATTCCATGCACTTGTCTATCAAACAGTGGTCCATACTCCCAACCCTTTTGCATTGCGCCAGTGTGTAAAATCACAGGTAGCCATGGTGTCATGCTTTATATACGAAGTCTTCCCTCTCTCTGCCCCTTGTGTGCCCTTGGCCCCTTTTTACAGACTATTGCTCACAATCTCAGGTGTCCAGCGGCCGCATTC | |
| miR-7-XIST2-Mut sequence | CCGCTCGAGTAGGCACAATGGCAGTTATCAATGGTTTTCTCCCTCCCTGACCTTGTTAAGCAAGCGCCCCACCCCACCCTTAGTTTCCCATGGCATAATAAAGTATAAGCATTGGAGTATTCCATGCACTTGTCTATCAAACAGTGGTCCATACTCCCAACCCTTTTGCATTGCGCCAGTGTGTAAAATCACAGGTAGCCATGGTGTCATGCTTTATATACGAACCTGAGCCTCTCTCTGCCCCTTGTGTGCCCTTGGCCCCTTTTTACAGACTATTGCTCACAATCTCAGGTGTCCAGCGGCCGCATTC | |
| miR-7-XIST3 sequence | CCGCTCGAGGCCCTAGGATATAAAAATGATGTTATCATTATAGAGTGCTCACAGAAGGAAATGAAGTAATATAGGTGTGAGATCCAGACCAAAAGTCATTTAACAAGTTTATTCAGTGATGAAAACATGGGACAAATGGACTAATATAAGGCAGTGTACTAAGCTGAGTAGAGAGATAAAGTCCTGTCCAGAAGATACATGCTTCCTGGCCTGATTGAGGAGATGGAAAATTTTTGCAAAAAACAAGGTGTTGTGGTCTTCCATCCAGTTTCTTAAGTGCTGATGATAAAAGTGAATTAGACCCACCTTGACCTGGCCTACAGAAGTAAAGGAGTAAAAATAAATGCCTCAGGCGTGCTGCGGCCGCATTC | |
| miR-7-XIST3-Mut sequence | CCGCTCGAGGCCCTAGGATATAAAAATGATGTTATCATTATAGAGTGCTCACAGAAGGAAATGAAGTAATATAGGTGTGAGATCCAGACCAAAAGTCATTTAACAAGTTTATTCAGTGATGAAAACATGGGACAAATGGACTAATATAAGGCAGTGTACTAAGCTGAGTAGAGAGATAAAGTCCTGTCCAGAAGATACATGCTTCCTGGCCTGATTGAGGAGATGGAAAATTTTTGCAAAAATGAAGGTGTTGTGGATGAGCATCCAGTTTCTTAAGTGCTGATGATAAAAGTGAATTAGACCCACCTTGACCTGGCCTACAGAAGTAAAGGAGTAAAAATAAATGCCTCAGGCGTGCTGCGGCCGCATTC | |
| miR-92b-XIST sequence | CTCGAGGCCATTGCTGCTGAGTTCTGACTACCCAAGTTTCCTTCTCTTAAACAGTTGATATGCATAATTGCATATATTCATGGTTCTGTGCAATAAAAATGGATTCTCACCCCATCCCACCTTCTGTGGGATGTTGCTAACGAGTGCAGATTATTCAATAACAGCTCTTGAACAGTTAATTTGCACAGTTGCAATTGTCCAGAGTCCTGTCCATTAGAAAGGGACTCTGTATCCTATTTGCACGCTACAATGTGGGCTGATGCGGCCGC | |
| miR-92b-XIST-Mut sequence | CTCGAGGCCATTGCTGCTGAGTTCTGACTACCCAAGTTTCCTTCTCTTAAACAGTTGATATGCATAATTGCATATATTCATGGTTCTGTGCAATAAAAATGGATTCTCACCCCATCCCACCTTCTGTGGGATGTTGCTAACGAGTGCAGATTATTCAATAACAGCTCTTGAACAGTTAATTTGCACAGTTGCAATTGTCCAGAGTCCTGTCCATTAGAAAGGGACTCTGTATCCTATTTGCACGCTACAATGTGGGCTGATGCGGCCGC | |
| miR-7/92b-Slug 3’UTR sequence | CTCGAGGTGACGCAATCAATGTTTACTCGAACAGAATGCATTTCTTCACTCCGAAGCCAAATGACAAATAAAGTCCAAAGGCATTTTCTCCTGTGCTGACCAACCAAATAATATGTATAGACACACACACATATGCACACACACACACACACACCCACAGAGAGAGAGCTGCAAGAGCATGGAATTCATGTGTTTAAAGATAATCCTTTCCATGTGAAGTTTAAAATTACTATATATTTGCTGATGGCTAGATTGAGAGAATAAAAGACAGTAACCTTTCTCTTCAAAGATAAAATGAAAAGCACATTGCATCTTTTCTTCCTAAAAAAATGCAAAGATTTACATTGCTGCCAAATCATTTCAACTGAAAAGAACAGTATTGCTTTGTAATAGAGTCTGTAATAGGATTTCCCATAGGAAGAGATCTGCCAGACGCGAACTCAGGTGCCTTAAAAAGTATTCCAAGTTTACTCCATTACATGTCGGTTGTCTGGTTGCCATTGTTGAACTAAAGCCTTTTTTTGATTACCTGTAGTGCTTTAAAGTATATTTTTAAAAGGGAGGAAAAAAATAACAAGAACAAAACACAGGAGAATGTATTAAAAGTATTTTTGTTTTGTTTTGTTTTTGCCAATTAACAGTATGTGCCTTGGGGGAGGAGGGAAAGATTAGCTTTGAACATTCCTGGCGCATGCTCCATTGTCTTACTATTTTAAAACATTTTAATAATTTTTGAAAATTAATTAAAGATGGGAATAAGTGCAAAAGAGGATTCTTACAAATTCATTAATGTACTTAAACTATTTCAAATGCATACCACAAATGCAATAATACAATACCCCTTCCAAGTGCCTTTTTAAATTGTATAGTTGATGAGTCAATGTAAATTTGTGTTTATTTTTATATGATTGAATGAGTTCTGTATGAAACTGAGATGTTGTCTATAGCTATGTCTATAAACAACCTGAAGACTTGTGAAATCAATGTTTCTTTTTTAAAAAACAATTTTCAAGTTTTTTTTACAATAAACAGTTTTGATTTAAAATCTCGTTTGTATACTATTTTCAGAGACTTTACTTGCTTCATGATTAGTACCAAACCACTGTACAAAGAATTGTTTGTTAACAAGAAAAAAAGCGGCCGC | |
| miR-7/92b-  Slug 3’UTR -Mut sequence | CTCGAGGTGACGCAATCAATGTTTACTCGAACAGAATGCATTTCTTCACTCCGAAGCCAAATGACAAATAAAGTCCAAAGGCATTTTCTCCTGTGCTGACCAACCAAATAATATGTATAGACACACACACATATGCACACACACACACACACACCCACAGAGAGAGAGCTGCAAGAGCATGGAATTCATGTGTTTAAAGATAATCCTTTCCATGTGAAGTTTAAAATTACTATATATTTGCTGATGGCTAGATTGAGAGAATAAAAGACAGTAACCTTTCTCTTCAAAGATAAAATGAAAAGCACATTGTGTGCTTCAAAGGTAAAAAAATGCAAAGATTTACATTGCTGCCAAATCATTTCAACTGAAAAGAACAGTATTGCTTTGTAATAGAGTCTGTAATAGGATTTCCCATAGGAAGAGATCTGCCAGACGCGAACTCAGGTGCCTTAAAAAGTATTCCAAGTTTACTCCATTACATGTCGGTTGTCTGGTTGCCATTGTTGAACTAAAGCCTTTTTTTGATTACCTGTAGTGCTTTAAAGTATATTTTTAAAAGGGAGGAAAAAAATAACAAGAACAAAACACAGGAGAATGTATTAAAAGTATTTTTGTTTTGTTTTGTTTTTGCCAATTAACAGTATGTGCCTTGGGGGAGGAGGGAAAGATTAGCTTTGAACATTCCTGGCGCATGCTCCATTGTCTTACTATTTTAAAACATTTTAATAATTTTTGAAAATTAATTAAAGATGGGAATAAGTGCAAAAGAGGATTCTTACAAATTCATTAATGTACTTAAACTATTTCAAATGCATACCACAAAATTGCAGATACAATACCCCTTCCAAGTGCCTTTTTAAATTGTATAGTTGATGAGTCAATGTAAATTTGTGTTTATTTTTATATGATTGAATGAGTTCTGTATGAAACTGAGATGTTGTCTATAGCTATGTCTATAAACAACCTGAAGACTTGTGAAATCAATGTTTCTTTTTTAAAAAACAATTTTCAAGTTTTTTTTACAATAAACAGTTTTGATTTAAAATCTCGTTTGTATACTATTTTCAGAGACTTTACTTGCTTCATGATTAGTACCAAACCACTGTACAAAGAATTGTTTGTTAACAAGAAAAAAAGCGGCCGC | |

**Supplementary Table S5. The primers for ChIP-PCR**

| Gene name | Forward primers(5’-3’) | Reverse primers(5’-3’) |
| --- | --- | --- |
| CD44-1 | AGAAGTCCTGGCATGGTTCC | CCATCTTTCCTACCCAGCAGA |
| CD44-2 | GAGAGGTGCCCATTCACACT | CAAGTCCACATGGCTTGAATGA |
| CD44-3 | CAAGACCTCGCCCTCTCTCC | AGGCTGTAAATAATCGGGGCTG |
| ESA-site1 | ACTGGAGTGCAGCGGTTTGGT | AGGTGGGCAGATCCGAGGTCA |
| ESA-site2 | AATCATCTCGGTTCTCAGTAT | CTAAAAGATACAAAATTAGCC |
| GAPDH | TACTAGCGGTTTTACGGGCG | TCGAACAGGAGGAGCAGAGAGCGA |

**Supplementary** **Table S6. The primer and probe sequences**

| **Gene/probe** | **Sequences(5’-3’)** |
| --- | --- |
| Probe(XIST1)1 | GAGCCCCACAGAAAGTAATC |
| Probe(XIST1)2 | ATGACTTTTGGTCTGGATCT |
| Probe(XIST1)3 | TGTATCTTCTGGACAGGACT |
| Probe(XIST1)4 | TAATTCACTTTTATCATCAG |
| Probe(XIST1)5 | TTCCACATAAAAGATGCTTT |
| Probe(XIST2)1 | TATACTTTATTATGCCATGG |
| Probe(XIST2)2 | ACACCATGGCTACCTGTGAT |
| Probe(XIST2)3 | CTGCAAATATGGACACCTGA |
| Probe(XIST2)4 | AAGAGAAAGGGCCTTGTCTG |
| Probe(XIST2)5 | TGGGTAGTCAGCATACTCAG |
| Probe(XIST3)1 | GCACCTTGATTGTCCAAACG |
| Probe(XIST3)2 | GCAAAAGGGGTCTGAGAGTA |
| Probe(XIST3)3 | GAAGGCTTCCTTGTCTAATA |
| Probe(XIST3)4 | ATAATTGTGCACCTTGATTG |
| Probe(XIST3)5 | AGGGCATCTGAGAGTAGGAC |
| Probe(Control) | TTCTCCGAACGTGTCACGT |
| XIST1 | F：GCTCGGAACTACATGCCCTA |
|  | R：TCAATCAGGCCAGGAAGCAT |
| XIST2 | F：CTGTCTCCCTCTTCCCTTCC |
|  | R：GCGAAAGGAAGTAGAGGGGT |
| XIST3 | F：ACCACTTTGCCCATTCCATC |
|  | R：AAAGGAAGACTGGGAGTGGG |
| Gapdh | F: AGGTCGGTGTGAACGGATTTG |
|  | R: GGGGTCGTTGATGGCAACA |
| miR-7-5p | F:TGTTGTTTTAGTGATCAGAAGGT |
| U6 | F:TGACACGCAAATTCGTGAAGCGTTCC |
